# Dynamic multifractal characteristics of acoustic emission about composite samples with different stress loading and unloading conditions

**DOI:** 10.1038/s41598-024-57839-9

**Published:** 2024-03-29

**Authors:** Yuben Liu, Xiangxi Meng

**Affiliations:** 1 College of Safety and Environmental Engineering, Shandong University of Science and Technology, Qingdao, 266590 China; 2 College of Geosciences and Surveying Engineering, China University of Mining and Technology (Beijing), Beijing, 100083 China; 3 Shandong Energy Group, Jinan, 250101 China

**Keywords:** Composite samples, Stress loading and unloading conditions, Multifractal characteristics, Acoustic emission characteristics, Civil engineering, Natural hazards, Petrology

## Abstract

Studying the failure characteristics of the common composite strata structure in western China is essential for evaluating stope stability and predicting coal mine dynamic disasters. To investigate the influence of different stress loading and unloading conditions on the instability characteristics of composite samples, three triaxial loading and unloading test schemes simulating different in-situ mining depths were designed. Complex triaxial tests were conducted on 12 sets of composite samples, and the bearing capacity, acoustic emission (AE) parameters and dynamic multifractal characteristics of the samples under different stress loading or unloading conditions were analyzed. The results indicate that samples tested by stress schemes simulating greater mining depths exhibit less damage, and the failure mode is a tensile-shear mixed failure, but the tensile failure is the main failure mode. The multifractal spectral parameters $\Delta \alpha$ of AE time series during the failure of composite samples tested with triaxial loading and unloading schemes simulating different mining depths show a decreasing trend in $\Delta \alpha$ values with increasing mining depth, while the change rules of $\Delta f\left( \alpha \right)$ values are the opposite. The multifractal parameter changes degree in four-layer rock structure composite samples under different stress conditions are lower than those in three-layer rock structure composite samples, indicating that the microcrack propagation process in the three-layer composite sample is more complex, resulting in higher levels of damage. The dynamic change of multifractal parameters $\Delta \alpha$ and $\Delta f\left( \alpha \right)$ during different stress loading and unloading stages reflects the influence of axial pressure or confining pressure changes on crack propagation in composite samples. Compared to the initial stress stage, the non-uniformity of AE signals increases in the residual stress stage, and the proportion of large signals becomes more prominent, signifying a complex micro-fracture process in the composite samples.

## Introduction

**Figure 1:**
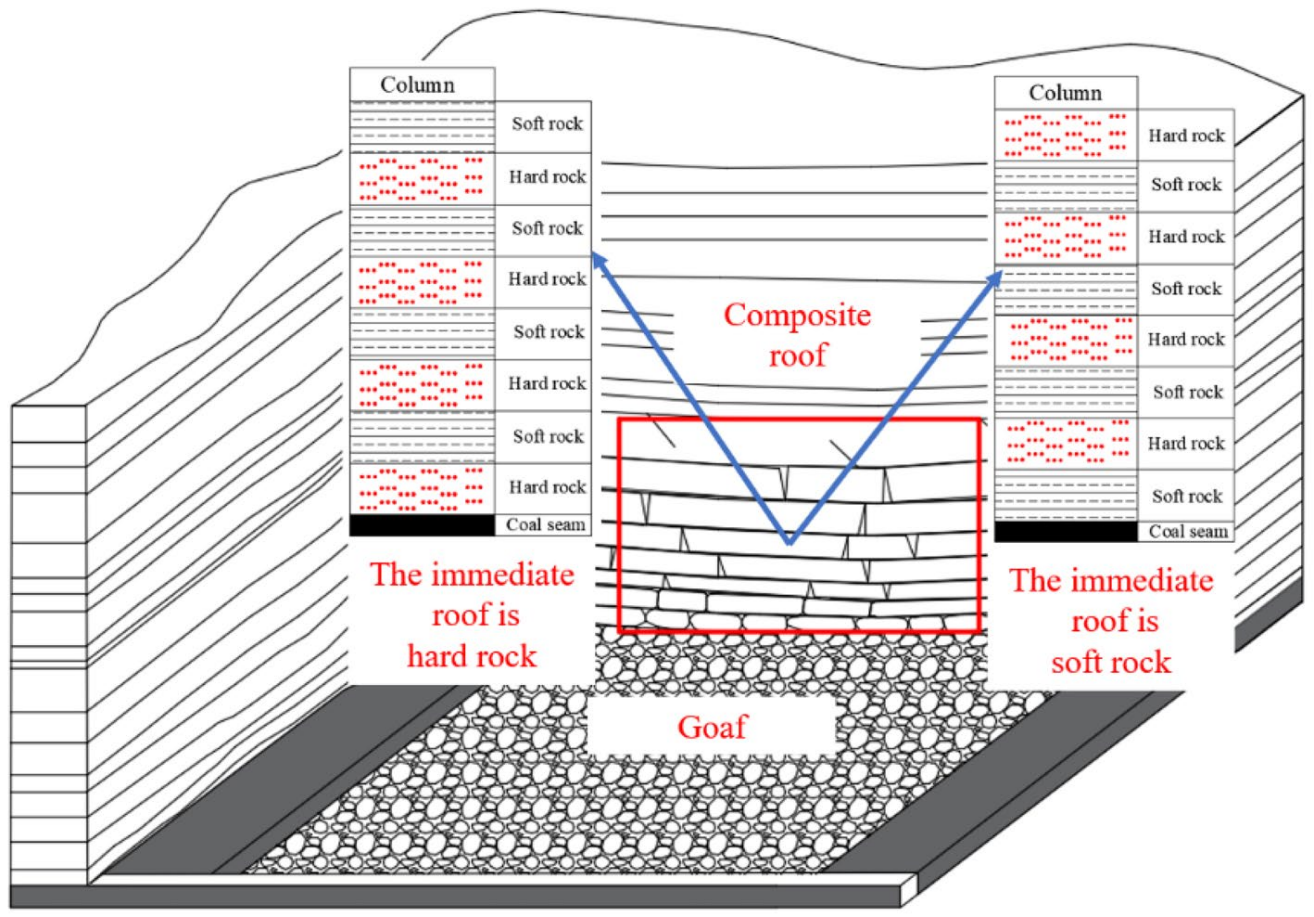
Strata structural characteristics of composite roof.

The composite roof is a common rock layer structure in Chinese coal-bearing formations, characterized by the interactive distribution of soft and hard rock layers^1–3^ (see the Fig. 1). Due to the tendency of soft rocks to undergo plastic failure and hard rocks to undergo brittle failure^4,5^, coupled with the influence of interfaces between rock layers on the overall stability^6,7^, the failure mode of composite roof rock strata is particularly complex. Therefore, it is an important challenge to explore the failure form of composite roof and the coordinated deformation law between rock strata.

Waterproof and sand-proof coal pillars will be set up in the process of coal mining^8,9^. To study overall stability of coal and rock formed by coal pillars and roof and floor strata, researchers have designed various combined samples^10^, such as roof strata-coal pillars^11^, coal pillars-floor strata^12^, roof strata-coal pillars-floor strata^13^ to simulate the structure between strata and coal pillars. Laboratory tests and numerical simulation methods were used to study the mechanical properties and failure behavior of coal-rock composite samples, as well as the influence of coal-rock height ratio^14^, stress loading path^15^, loading rate^16,17^, confining pressure^18^, rock strength^19^ and other factors on coal-rock composite samples. In addition, Liu et al. believed that compared with single coal rock, the instability and failure process of combined coal rock samples can better reflect the instability and failure process of coal rock in the actual mining site^20^. The above research work provides research ideas for our research. Through the analysis of the structure of the composite roof strata, the composite roof of the coal seam is roughly divided into four categories, namely, soft rock-hard rock -soft rock structure, hard rock -soft rock -hard rock structure, soft rock -hard rock -soft rock -hard rock structure, hard rock -soft rock -hard rock -soft rock structure. According to the above four types of structures, four different rock composite samples were designed. The stability of the composite rock strata was analyzed by using the stress loading and unloading scheme that can simulate the actual mining situation on site to test the failure characteristics of the composite samples. In recent years, Xie et al. summarized the common characteristics of the abutment pressure distribution in front of the working face of three different mining modes (protective coal-seam mining, top-coal caving mining, and non-pillar mining, respectively). They suggest that as the mining face advances, the support pressure in the front of the working face begins to increase, while the radial stress gradually decreases^21,22^. After that, Chen et al. designed a stress loading and unloading scheme that can simulate the real coal mining site according to the evolution of the abutment pressure characteristics of the coal seam above the working face, and studied the crack propagation and damage behavior of sandstone in detail through experiments^23^. Building upon this, our research, considering the stress distribution in front of the working face during coal seam extraction, has designed three sets of triaxial loading and unloading test schemes to simulate different in-situ mining depths (simulating mining depths of 500, 600, and 700 m, respectively).

During the failure process of the sample, the sample releases a wealth of physical signals such as electromagnetic radiation^24^, acoustic emission (AE)^25^, charge signal^26^, thermal radiation^27^ and other signals to the outside. The AE signal refers to the weak acoustic signal released during the propagation and micro-fracture in the rock under the action of external stress^20^. These acoustic signals record the microscopic details of rock failure^28–30^. Therefore, using AE data, we can further analyze the deformation and fracture mechanism of rock. Although AE data can provide information about the internal deformation and evolution of cracks in rocks, it is often qualitative and lacks quantitative characterization. Fractal analysis can quantitatively describe the overall non-uniformity and irregularity, possessing self-similarity and scale invariance. The fractal dimension of AE signal can predict the failure state of rock. However, rock failure is a complex nonlinear process and exhibits inhomogeneity at different locations and scales^31,32^. Therefore, the traditional single fractal theory cannot fully describe the macroscopic mechanism of rock failure. Multifractal analysis is a tool for studying complex systems, which can reveal the inherent nonlinearity and scale correlation of the system^33,34^. Therefore, by analyzing the multifractal characteristics of the AE signal, the damage evolution and loading process of the sample can be reflected according to the dynamic changes of the multifractal spectrum parameters.

In this paper, the rock structure of composite roof, which is widely distributed in China, is taken as the research object. According to its structural characteristics, four groups of composite rock samples with different rock structure forms are designed. Considering the stress distribution characteristics of stope before and after coal seam mining, three kinds of triaxial loading and unloading test schemes are designed to simulate different mining depth conditions in the field. Complex conventional triaxial loading and unloading tests were carried out on 12 groups of composite samples. The originality and core objectives of this paper is to analyze the influence of stress loading and unloading conditions on the peak strength, failure mode and the dynamic multifractal characteristics of the AE time series of composite samples. Additionally, the multifractal parameters of composite samples with different stress conditions and the multifractal dynamic characteristics of the AE time series of composite samples at different stress stages were discussed. The results can provide support for the study of damage evolution rules and quantitative characterization of composite roof.

## Materials and methods

### Conventional triaxial loading and unloading test

#### Testing system and sample preparations

In the testing, the conventional triaxial loading and unloading test was carried out using the RMT-150B electro-hydraulic servo rock test system at room temperature of 27 °C. The test system uses a 1000 kN force sensor to measure the axial load, and the load accuracy is 1.0 × 10^−3^ kN. At the same time, a 5.0 mm displacement sensor is used to monitor the axial compression deformation. The confining pressure monitoring uses a 50 MPa confining pressure sensor. During the loading process of the sample, the AE information was synchronously monitored by the DS-5 type 8-channel AE detection and analysis system. The sampling frequency was set to 1 MHz, the threshold value was set to 50 dB, and the frequency of the AE sensor RS-2A was 150 kHz. During the monitoring process, the acquisition system records the amplitude, count, energy and frequency of AE in real time.

**Figure 2:**
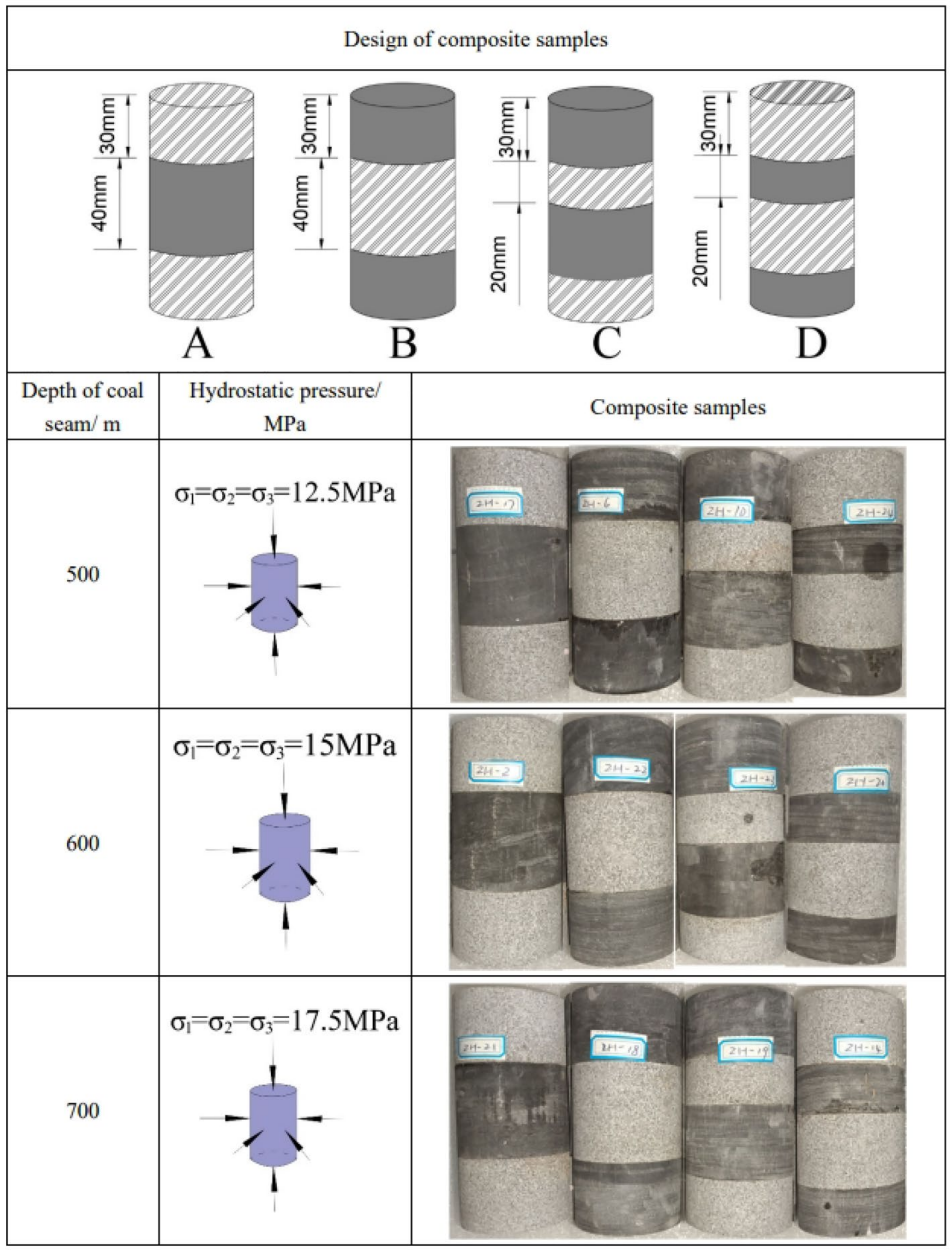
The design of composite samples.

Based on the typical structural characteristics of composite roof strata mentioned in the Introduction, a total of four representative sets of composite samples were designed for this experiment, labeled as A, B, C, and D, each comprising 3–6 samples. The samples were screened by calculating the multifractal parameters during the static hydrostatic pressure stage. The preparation of composite samples involves several steps. Initially, rock samples collected from the field are cut to the specified dimensions using a rock cutting machine. Subsequently, the rock surfaces are ground using a polishing machine, followed by wiping the samples with clean tissue. Based on research requirements, the combination ratios are determined, and epoxy resin is applied to bond the various components of the rock. The bonded samples are then wrapped in cling film and stored in foam boxes. The designed 4 sets of composite samples include two major categories: one with a three-layer rock layer structure, consisting of hard rock-soft rock-hard rock structure and soft rock-hard rock-soft rock structure (labeled as A and B, respectively); the other with a four-layer rock layer structure, consisting of hard rock-soft rock-hard rock-soft rock and soft rock-hard rock-soft rock-hard rock structures (labeled as C and D, respectively), as shown in Fig. 2. The A, B, C, D samples were subjected to triaxial compression tests under three different stress conditions at coal seam depths of 500 m (labeled as A-1, B-1, C-1, and D-1, static hydrostatic pressure of 12.5 MPa), 600 m (labeled as A-2, B-2, C-2, and D-2, static hydrostatic pressure of 15 MPa), and 700 m (labeled as A-3, B-3, C-3, and D-3, static hydrostatic pressure of 17.5 MPa). The soft-hard-soft structure indicates that the bottommost layer of the composite samples is composed of soft rock, arranged from bottom to top as soft rock-hard rock-soft rock. This design mimics different immediate roof lithologies and various rock layer combinations in composite roof strata.

#### Conventional triaxial loading and unloading test scheme

**Figure 3:**
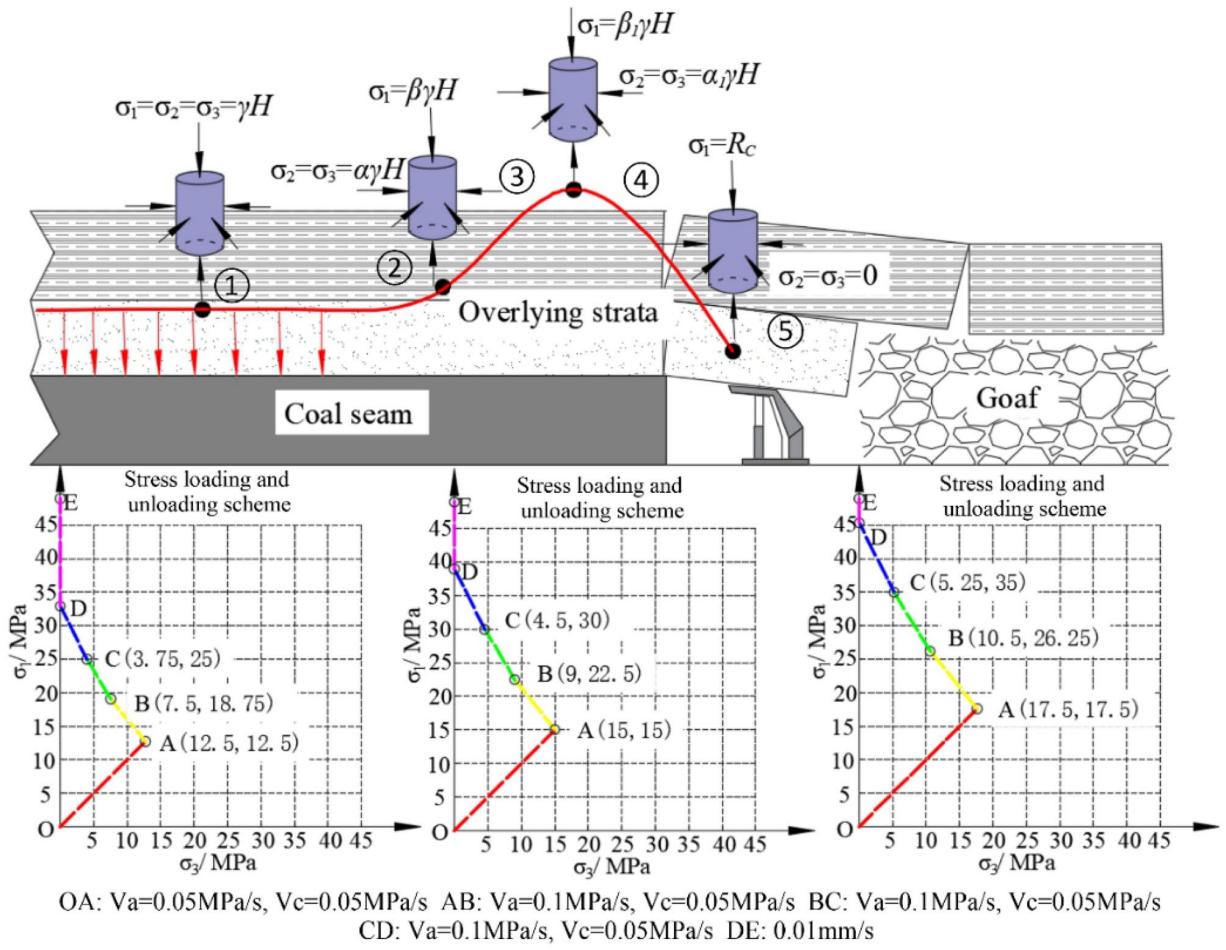
Stress distribution in front of the working face and stress loading and unloading scheme.

A substantial amount of research indicates that the stress distribution in the overlying strata in front of the coal face can be divided into five stages^21–23^, as illustrated in Fig. 3. These five stages are the hydrostatic pressure stage (①), the first axial pressure increase and confining pressure release stage (②), the second axial pressure increase and confining pressure release stage (③), the continuous release of confining pressure stage (④), and the complete release of confining pressure stage (⑤). In this paper, based on the stress distribution in front of the working face, three sets of triaxial loading and unloading test schemes were designed for different mining depths. According to these schemes, strength tests with varying gradient confining pressures were conducted on composite samples.

Scheme 1: The scheme simulates the stress conditions at a coal seam mining depth of 500 m. It is assumed that the vertical stress gradient *γ* is 25 kPa/m, so the vertical and horizontal stress levels are both about 12.5 MPa (OA) in the hydrostatic pressure stage. Stress concentration factors from the literature were used^21–23^, with stress coefficients selected as *α*=0.6, *β*=105, *α*_1_=0.3, *β*_1_=2.Therefore, with the mining of the working face, the stress distribution enters Stage ②, during which the confining pressure decreases to 7.5 MPa, and axial pressure increases to 18.75 MPa (AB). Progressing into Stage ③, the confining pressure further decreases to 3.75 MPa, and pressure increases to 25 MPa (BC). It is crucial to emphasize that during the transition from Stage ③ to Stage ④, where the confining pressure continues to decrease to zero, two cases may occur. The first case involves sample failure during the unloading of confining pressure. In this case, the sample’s strength can be directly obtained, and the triaxial loading and unloading test scheme only needs to follow three stages: OA-AB-BC. In such case, the sample is considered to have a lower strength, indicating that the rock structure lacks high bearing capacity, leading to advanced failure ahead of the working face during coal mining. The second case is when the sample does not fail as the confining pressure continues to decrease. In this case, to determine the sample’s strength, it is necessary to further increase axial pressure while simultaneously decreasing confining pressure (CD) until reaching a uniaxial compression state (DE), ultimately resulting in sample failure. The strength obtained in the second case is higher, indicating that this type of rock structure has greater bearing capacity and does not undergo premature failure during coal seam mining.

Scheme 2 The scheme is designed to simulate the stress conditions of coal seam mining depth of 600 m. During the hydrostatic pressure stage, the vertical and horizontal stresses are approximately 15 MPa each (OA). With the mining of the working face, the stress distribution enters Stage ②, during which the confining pressure decreases to 9 MPa, and axial pressure increases to 22.5 MPa (AB). Progressing into Stage ③, the surrounding pressure further decreases to 4.5 MPa, and axial pressure increases to 30 MPa (BC). The stress variations in other stages follow the loading and unloading patterns of Scheme 1, and the stress values during different stages of loading and unloading are showed in Fig. 3.

Scheme 3: Consistent with the stress variations described above, the Scheme 3 simulates the stress conditions of coal seam mining depth of 700 m. During the hydrostatic pressure stage, the vertical and horizontal stresses are approximately 17.5 MPa each (OA). As the mining face advances, the stress distribution enters Stage ②, during which the confining pressure decreases to 10.5 MPa, and axial pressure increases to 26.25 MPa (AB). Progressing into Stage ③, the confining pressure further decreases to 5.25 MPa, and axial pressure increases to 35 MPa (BC). The stress variations in other stages follow the loading and unloading patterns of Scheme 1, and the stress values during different stages of loading and unloading are showed in Fig. 3.

Because the stress environment of rock strata in the whole mining process is complex and changeable, it is found that the mixed loading mode (stress–strain loading) is more suitable for this study after many tests when selecting the loading mode. The advantage of mixed loading is that it combines the advantages of the two loading methods. Through the stress loading stage, the stress–strain characteristics and strength of the material can be directly measured, and in the strain loading stage, a constant strain test can be performed to avoid damage caused by excessive loading. This loading method can more fully understand the mechanical properties of the material. The loading rate of confining pressure in the hydrostatic pressure stage is 0.05 MPa/s, and the loading rate of axial pressure is 0.1 kN/s (stress loading). The loading rate of axial compression in the unloading stage is 0.2 kN/s, and the unloading rate of confining pressure is 0.05 MPa/s (stress loading). The uniaxial compression stage is controlled by displacement, and the displacement loading rate is 0.01 mm/s (strain loading).

### The calculation principle of multifractal

The fracture progression in rock is often accompanied by the compaction of internal primary cracks, along with the generation and expansion of new cracks. These processes exhibit discontinuity, giving rise to discrete and nonlinear AE signals. As a result, the AE signal displays a diverse self-similarity within its fractal structure. However, conventional fractal methods may fall short in fully capturing the comprehensive failure process of the rock. To enhance our understanding of the deformation and failure processes in composite coal rock samples, a multifractal calculation method was employed to deconstruct and analyze the evolving patterns of AE signals. Within the multifractal spectrum calculation method, the box dimension calculation approach stands out as a statistically grounded method. It simplifies the complexity of the analysis while effectively uncovering the deformation and failure processes of composite samples. This method furnishes valuable insights for further research in the field.

In this calculation method, by calculating the partition function (Eq. (1)), we can understand the distribution and variation of AE signals at different scales, so as to judge whether the research object has multifractal characteristics^33,34^.

1 $$ X_{q} (r) = \sum\limits_{i = 1}^{N} {\left[ {P_{i} (r)} \right]^{q} } $$

where $P_{i} (r)$ is the cracks probability function; $q$ is the order moment (Theoretically, $q$ can be any real number. But in the actual calculation, when $\left| q \right|$ reaches a certain value, the multifractal spectrum tends to be stable, and the larger $q$ value will not affect the multifractal spectrum).

The relationship between the partition function and the partition scale $r$ is shown in Eq. (2). The equation is a prerequisite to prove that the AE signal has fractal characteristics in the corresponding scale range.

2 $$ X_{q} (r) \propto r^{\tau (q)} $$

where $\tau (q)$ is the quality index.

According to the above relationship, the quality index $\tau (q)$ can be obtained by taking logarithms on both sides of Eq. (2).

3 $$ \tau (q) = \mathop {\lim }\limits_{r \to 0} \frac{{\ln X_{q} (r)}}{\ln r} $$

If the figure of the $\ln X_{q} (r) - \ln r$ is a series of straight lines with different slopes, it indicates that the research object has multifractal characteristics, and its multifractal spectrum and its characteristic parameters can be further calculated.

The generalized fractal dimension $D(q)$ is a fractal dimension that has different meanings with the value of order moment $q$, and its equation is related to the mass index and order moment.

4 $$ D_{q} = \left\{ {\begin{array}{*{20}c} {\tau (q)/(q - 1)} & {\left( {q \ne 1} \right)} \\ {\tau (1)} & {\left( {q{ = }1} \right)} \\ \end{array} } \right. $$

For multifractal, the quality index $\tau (q)$ and the generalized fractal dimension $D(q)$ of multifractal describe the characteristics of multifractal as a whole, while the singularity index $\alpha$ and the multifractal spectral function $f(\alpha )$ can describe the local characteristics of multifractal, and the singularity index $\alpha$ and the multifractal spectral function $f(\alpha )$ satisfy Legendre transformation.

5 $$ \left\{ {\begin{array}{*{20}l} {\alpha (q) = \frac{d\tau (q)}{{dq}}} \hfill \\ {f(\alpha ) = q \cdot \alpha (q) - \tau (q)} \hfill \\ \end{array} } \right. $$

## Results and discussions

### The failure characteristics of composite samples under different stress loading and unloading conditions

**Figure 4:**
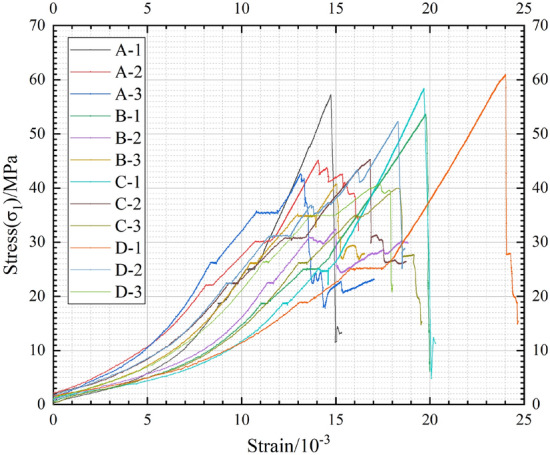
The stress–strain curves of composite samples with different stress schemes.

Figure 4 displays the stress–strain curves of four groups of composite samples A, B, C and D designed in Section “Conventional triaxial loading and unloading test” using stress schemes simulating different mining depths. In the figure, A-1 indicates that group A samples are loaded by stress Scheme 1, A-2 indicates that group A samples are loaded by stress Scheme 2, and A-3 indicates that group A samples are loaded by stress Scheme 3. The naming rules of B, C and D are consistent with A. With the exception of the soft rock-hard rock-soft rock (B group) rock structure, the other three types of composite samples, under the designed stress loading and unloading schemes, exhibit a trend of decreasing strength with increasing mining depth. For example, A-1 (57.2 MPa) > A-2 (45.0 MPa) > A-3 (42.6 MPa), C-1 (58.3 MPa) > C-2 (45.1 MPa) > C-3 (39.9 MPa), D-1 (60.9 MPa) > D-2 (52.2 MPa) > D-3 (40.8 MPa). This result needs to be further discussed, as outlined below: Increasing mining depth corresponds to an increase in hydrostatic pressure, which implies a larger initial confining pressure on composite samples. A higher initial confining pressure generally enhances the compressive strength of rocks^35^. This contradicts the experimental results of this study. Through an analysis of the designed loading and unloading schemes in this study, it was identified that the possible reason for the above results is mainly the difference of deviatoric stress ($\sigma_{1} - \sigma_{3}$) in different mining stages for different mining depths. In Scheme 1, during the first axial pressure increase and surrounding pressure release stage, the deviatoric stress ($\sigma_{1} - \sigma_{3}$) increases from 0 to 11.25 MPa. As the mining face continues to advance, in the second axial pressure increase and confining pressure release stage, the deviatoric stress ($\sigma_{1} - \sigma_{3}$) further increases from 11.25 to 21.25 MPa. In Scheme 2, during the first axial pressure increase and confining pressure release stage, the deviatoric stress ($\sigma_{1} - \sigma_{3}$) increases from 0 to 13.5 MPa, and with the continued mining of the working face, in the second axial pressure increase and confining pressure release stage, the deviatoric stress ($\sigma_{1} - \sigma_{3}$) further increases from 13.5 to 25.5 MPa. In Scheme 3, during the first axial pressure increase and confining pressure release stage, the deviatoric stress ($\sigma_{1} - \sigma_{3}$) increases from 0 to 15.75 MPa, and with the continued advancement of the working face, in the second axial pressure increase and confining pressure release stage, the deviatoric stress ($\sigma_{1} - \sigma_{3}$) further increases from 15.75 to 29.75 MPa. Therefore, despite greater mining depth resulting in a larger initial confining pressure, the increase in deviatoric stress ($\sigma_{1} - \sigma_{3}$) during the axial pressure increase and confining pressure release stage leads to a reduction in the ultimate compressive strength of the samples. This result is consistent with the greater degree of rock damage in deep mining compared with shallow coal seam mining. Another possible reason is the influence of the composite rock structure. The larger initial confining pressure inhibits cracks generated in soft rock from extending into the hard rock layers. As a result, the damage primarily occurs in the soft rock, leading to an overall reduction in strength. Furthermore, the initial pressure has a disturbance to the samples, and it is also possible that the high initial confining pressure and axial pressure affect the integrity of the samples.

Additionally, within Group B, B-1 (53.6 MPa) > B-3 (40.7 MPa) > B-2 (32.4 MPa), and the reason for this outcome may be attributed to the rock layer structure. The rock layer structure of the samples in Group B is soft rock-hard rock-soft rock. The larger initial confining pressure suppresses the expansion of cracks in the soft rock, enhancing the load-bearing capacity of the soft rock. Overall, samples with a four-layer rock structure loaded with stress Scheme 3, simulating a mining depth of 700 m (Groups C and D), exhibit lower strength compared to samples with a three-layer rock structure (Groups A and B). Conversely, samples with a four-layer rock structure loaded with stress Schemes 1 and 2, simulating mining depths of 500 and 600 m (Groups C and D), demonstrate higher strength than samples with a three-layer rock structure (Groups A and B). This indicates that with increasing mining depth, the load-bearing capacity of different rock layer structures undergoes changes.

### AE characteristics of failure process of composite samples with different stress loading and unloading conditions

**Figure 5:**
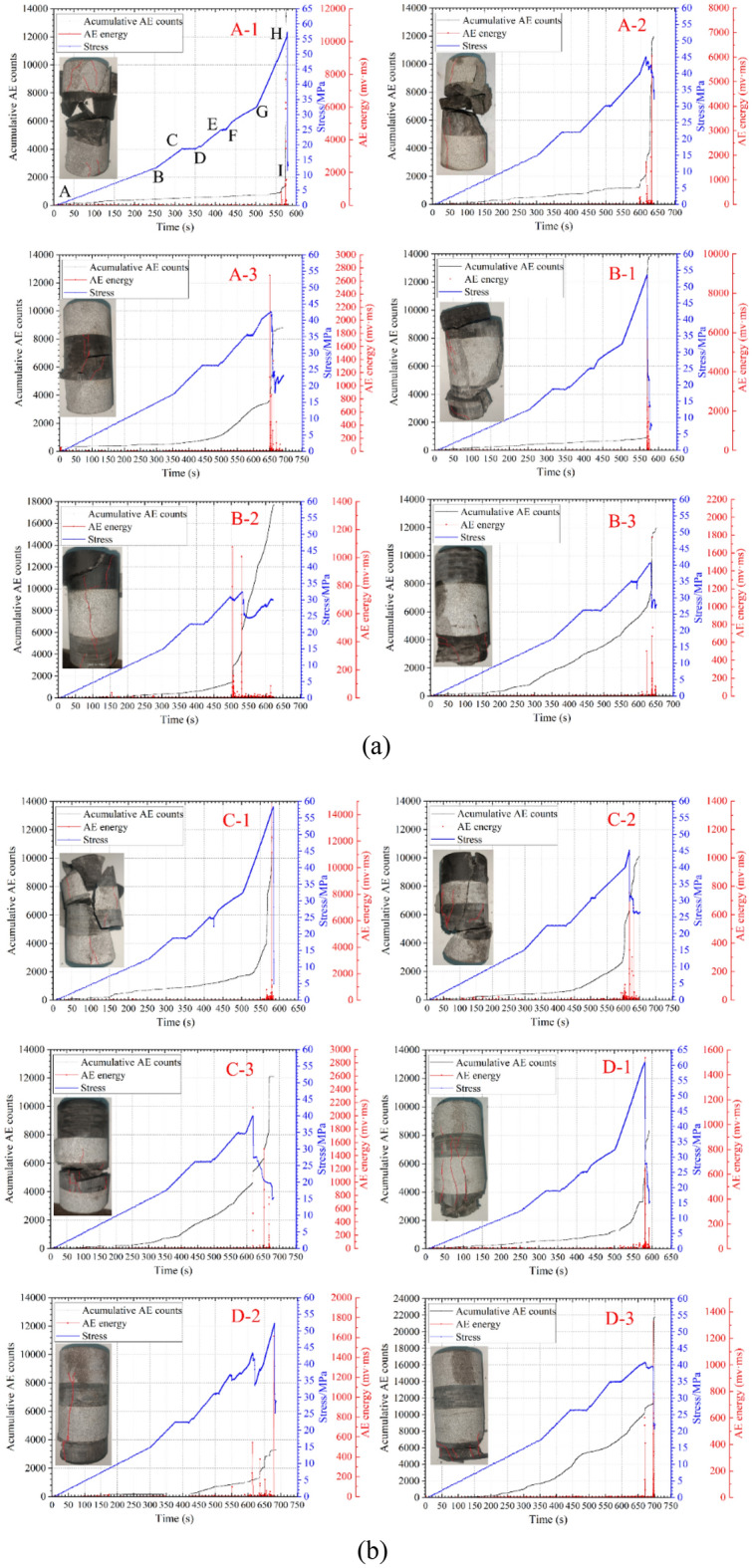
The AE test results of composite samples with different stress schemes, (**a**) Three-layer rock structure; (**b**) Four-layer rock structure.

The failure of composite samples releases AE signals outward, and these AE signals propagate through the rock and are detected by surface sensors. Due to the large number of test samples, the results for one sample from each group of composite samples are provided. Figure 5 illustrates the variations over time in loading stress, cumulative number of AE events, and AE energy during the deformation process of the composite samples. It can be seen from Fig. 5 that the AE signal gradually increases with the loading of external stress. For the three-layer rock samples (groups A and B) using the stress Scheme 1, the AE signals mainly exhibit two phases: slow increase and rapid increase. As the mining depth increases (stress Schemes 2 and 3), the AE signals gradually transition into three phases: slow increase, transitional increase, and rapid increase. For the four-layer rock samples (groups C and D), the variation in AE signals during the loading of external stress follows a similar pattern, with three phases: slow increase, transitional increase, and rapid increase. It is obvious that with the increase of mining depth, the damage degree of the sample shows a decreasing trend. The main reason is that with the increase of mining depth, the initial confining pressure in the stress loading and unloading scheme increases, and the larger confining pressure inhibits the expansion of the internal cracks of the composite sample. The failure mode of all samples is tensile-shear mixed failure, but tensile failure is the main failure mode. In addition, in a composite sample, the hard rock layer shows tensile failure while the soft rock layer mainly shows tensile-shear mixed failure.

### Multifractal characteristics of AE time series of composite samples

**Figure 6:**
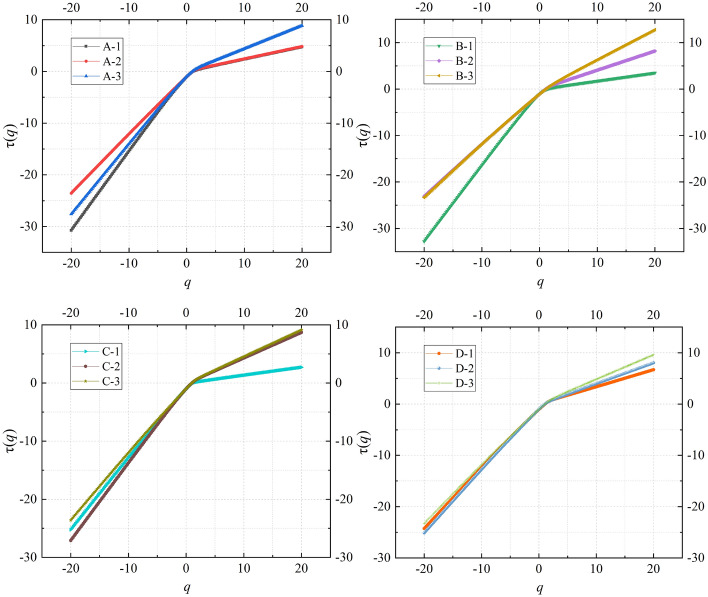
The curve of mass index function.

$\tau (q)$ in Eq. (3) is a mass index function, which is the characteristic function of fractal behavior. If $\tau (q)$ is linear with respect to $q$, then the subject of study exhibits single fractal characteristics. However, if $\tau (q)$ is nonlinear, especially appearing as a convex function concerning $q$, then the research subject possesses multifractal characteristics, and the more pronounced the nonlinearity, the greater the strength of the multifractal. It can be seen from Fig. 6 that the mass index function $\tau (q)$ of the AE signals of all composite samples shows the characteristics of convex function with the increase of $q$, which indicates that the AE signals of the time series have multifractal characteristics. In addition, when $q$ is a positive number, the value of the mass index function $\tau (q)$ increases with the increase of the mining depth (e.g., A-1 < A-2 < A-3, where the mass index function of the AE signal of the sample loaded by the stress Scheme 3 with the simulated mining depth of 700 m is the largest, and the mass index function of the AE signal of the sample loaded by the stress Scheme 1 with the simulated mining depth of 500 m is the smallest).

**Figure 7:**
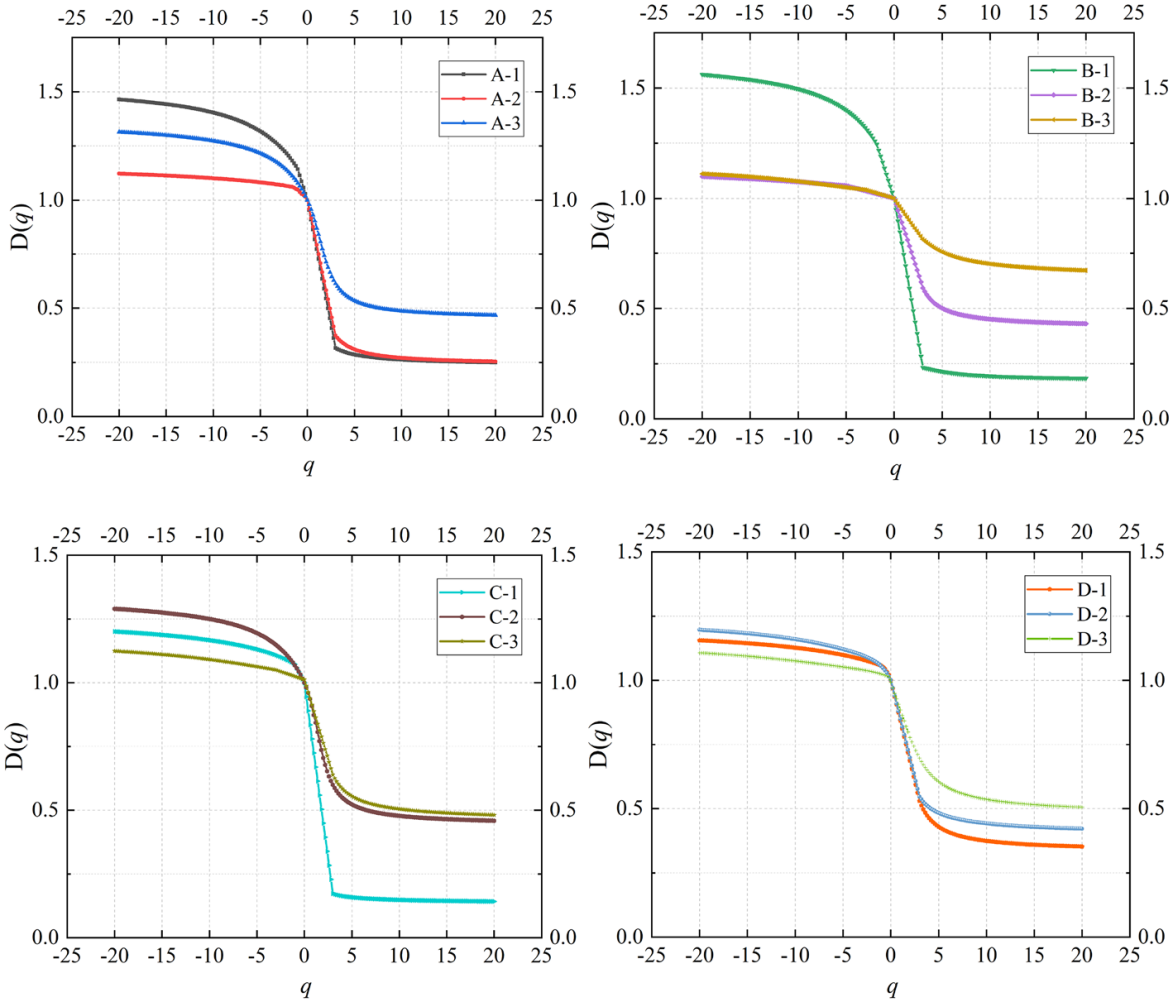
The curve of generalized fractal dimension.

In Eq. (4), the generalized fractal dimension $D(q)$ can determine the range of values for $q$. When the absolute value of $q$ exceeds a certain threshold, the value of $D(q)$ tends to stabilize. As observed in Fig. 7, when the absolute value of $q$ is greater than 20, $D(q)$ gradually tends to be a straight line. Therefore, the chosen range for $q$ in this study is set between − 20 and + 20. Additionally, in alignment with the results in Fig. 6, when $q$ is a positive value, the greater the depth of coal seam mining, the greater the value of the generalized fractal dimension $D(q)$ of AE under stress conditions.

After determining that the AE signal has multifractal characteristics in the time series, according to the definition of Eq. (5), this paper uses $\alpha \sim f\left( \alpha \right)$ to describe the multifractal characteristics of the AE time series. Among them, $\alpha$ represents different subsets of AE signals, the subset corresponding to $\alpha_{\max }$ represents small signals in AE time series, and the subset corresponding to $\alpha_{\min }$ represents large signals in AE time series. The width $\Delta \alpha = \alpha_{\max } - \alpha_{\min }$ of multifractal spectrum can reflect the difference of signals of different sizes in AE event series. The wider the multifractal spectrum is, the larger the corresponding value of $\Delta \alpha$ is, the more obvious the multifractal characteristics are, the larger the gap between the AE signals, the greater the inhomogeneity between the signals, and the more severe the fluctuation of the signal distribution is. The size of $f\left( \alpha \right)$ represents the frequency singularity of the AE signal subset during the whole loading process. $\Delta f\left( \alpha \right){ = }f\left( {\alpha_{\max } } \right) - f\left( {\alpha_{\min } } \right)$ is a measure of the difference in the number of large and small signals. $\Delta f\left( \alpha \right)$ > 0 indicates that small signal is dominant, and $\Delta f\left( \alpha \right)$ < 0 indicates that large signal is dominant. When $\Delta \alpha$ is the same, the smaller $\Delta f\left( \alpha \right)$ is, the higher the proportion of large signals is, and the more complex the micro-fracture process of the composite sample is.

**Figure 8:**
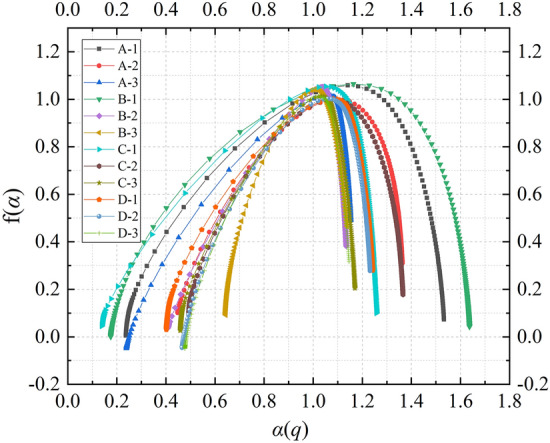
Dynamic multifractal spectra of different samples with different stress schemes.

Figure 8 displays the multifractal spectra of AE signals for 12 groups of composite samples with different stress loading and unloading conditions simulating different mining depths conditions. Overall, the multifractal spectra for all composite samples exhibit a right-hook shape, indicating that the macroscopic generation mechanism of AE events is similar, with a higher frequency of occurrence for small events compared to large events within the AE signals. This is attributed to the gradual accumulation of cracks in the composite samples, where microcracks accumulate to a certain extent before forming macroscopic cracks, leading to sample failure. However, there are notable differences in the parameters $\Delta \alpha$ and $\Delta f\left( \alpha \right)$ of the multifractal spectra, reflecting variations in the microscale complexity of the AE events. This diversity arises from differences in the structures of the composite samples, variations in initial damage levels, and the uneven distribution of internal microcracks, along with differing conditions for crack friction, slip, and extension. To quantitatively describe the effect of different stress loading and unloading methods on the unevenness of AE signals and the proportionality between small and large signals, this study further calculates and compares the parameters of the multifractal spectra, as shown in Table 1 From the table, it can be observed that the value of the multifractal spectrum parameter $\Delta \alpha$ is significantly influenced by the mining depth. Specifically, the multifractal parameter $\Delta \alpha$ of the AE time series of the sample loaded by the stress Scheme 3 with the simulated mining depth of 700 m is the smallest, and the multifractal parameter $\Delta \alpha$ of the AE time series of the sample loaded by the stress Scheme 1 with the simulated mining depth of 500 m is the largest. This shows that the more uniform the AE signal is, the lower the fluctuation of the signal is when the stress scheme with the larger mining depth is used to load the sample. Combined with the failure mode of the sample in Fig. 5, it can be seen that Scheme 3 has a large confining pressure in the hydrostatic pressure stage, and the large confining pressure inhibits the generation of penetrating cracks. The failure of the sample mainly occurs in the relatively weaker rock layers, so the AE signal is relatively uniform. The change rules of multifractal spectrum parameter $\Delta f\left( \alpha \right)$ is opposite to that of $\Delta \alpha$. The overall performance is that the value of multifractal parameter $\Delta f\left( \alpha \right)$ of the AE time series of the samples loaded by the stress Scheme 3 with the simulated mining depth of 700 m is the largest, and the value of multifractal parameter $\Delta f\left( \alpha \right)$ of the AE time series of the samples loaded by the stress Scheme 1 with the simulated mining depth of 500 m is the largest. The variation pattern of the multifractal spectrum parameter $\Delta f\left( \alpha \right)$ is opposite to that of $\Delta \alpha$, showing an overall trend where samples loaded using stress Scheme 3 (simulating mining depth of 700 m) exhibit the largest value for the multifractal parameter $\Delta f\left( \alpha \right)$, while those loaded using stress Scheme 1 (simulating mining depth of 500 m) have the smallest value for the multifractal parameter $\Delta f\left( \alpha \right)$. This is because $\Delta f\left( \alpha \right)$ reflects the proportion difference between small and large signals, with smaller $\Delta f\left( \alpha \right)$ values indicating a higher proportion of large signals and a more complex micro-fracture process in the composite samples. Additionally, the study provides the ratio of multifractal parameters with different stress schemes. From the table, it can be observed that the $\Delta f\left( \alpha \right)$ value for D-3 is 1.448 times that of D-1, the $\Delta f\left( \alpha \right)$ value for C-3 is 3.236 times that of C-1, and the $\Delta f\left( \alpha \right)$ value for B-3 is 8.905 times that of B-1. Similarly, the $\Delta f\left( \alpha \right)$ value for A-3 is 7.765 times that of A-1. This indicates that the variation in multifractal parameters for composite samples with a four-layer rock structure (Groups C and D) is lower than that of composite samples with three-layer rock structure (Groups A and B) with different loading schemes. Three-layer composite samples exhibit a more complex micro-fracture process and higher levels of damage. The values of $\Delta \alpha$ for A-2 and A-3 are 0.705 times that of A-1, while for B-2 and B-3, the values of $\Delta \alpha$ are 0.495 and 0.338 times that of B-1, respectively. Similarly, for C-2 and C-3, the values of $\Delta \alpha$ are 0.779 and 0.636 times that of C-1, and for D-2 and D-3, the values of $\Delta \alpha$ are 0.911 and 0.794 times that of D-1. Overall, there is a consistent trend of decreasing values of $\Delta \alpha$ with increasing mining depth. The trend suggests that when samples are subjected to stress loading schemes associated with greater mining depths, crack propagation tends to be more uniform, resulting in lower levels of damage.

**Table 1:** Multifractal parameters of different samples with different stress schemes.

No	$\Delta \alpha$	$\Delta f\left( \alpha \right)$	$\frac{{\Delta \alpha \left( {\text{A - n}} \right)}}{{\Delta \alpha \left( {\text{A - 1}} \right)}}$	$\frac{{\Delta \alpha \left( {\text{B - n}} \right)}}{{\Delta \alpha \left( {\text{B - 1}} \right)}}$	$\frac{{\Delta \alpha \left( {{\text{C}} - {\text{N}} } \right)}}{{\Delta \alpha \left( {\text{C - 1}} \right)}}$	$\frac{{\Delta \alpha \left( {{\text{D}} - {\text{N}} } \right)}}{{\Delta \alpha \left( {\text{D - 1}} \right)}}$
			$\frac{{\Delta f\left( \alpha \right)\left( {\text{A - n}} \right)}}{{\Delta f\left( \alpha \right)\left( {\text{A - 1}} \right)}}$	$\frac{{\Delta f\left( \alpha \right)\left( {\text{B - n}} \right)}}{{\Delta f\left( \alpha \right)\left( {\text{B - 1}} \right)}}$	$\frac{{\Delta f\left( \alpha \right)\left( {\text{C - n}} \right)}}{{\Delta f\left( \alpha \right)\left( {\text{C - 1}} \right)}}$	$\frac{{\Delta f\left( \alpha \right)\left( {\text{D - n}} \right)}}{{\Delta f\left( \alpha \right)\left( {\text{D - 1}} \right)}}$
A-1	1.297	0.068	1.000 1.000			
A-2	0.915	0.206	0.705 3.029			
A-3	0.914	0.528	0.705 7.765			
B-1	1.462	0.042		1.000 1.000		
B-2	0.723	0.329		0.495 7.833		
B-3	0.494	0.374		0.338 8.905		
C-1	1.120	0.055			1.000 1.000	
C-2	0.873	0.076			0.779 1.382	
C-3	0.712	0.178			0.636 3.236	
D-1	0.841	0.250				1.000 1.000
D-2	0.766	0.323				0.911 1.292
D-3	0.668	0.362				0.794 1.448

**Figure 9:**
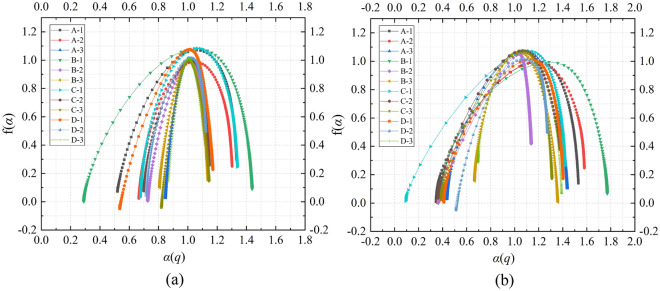
Multifractal spectrum of ringing cumulative count before and after mutation: (**a**) Multifractal spectrum of ringing cumulative count before mutation (**b**) Multifractal spectrum of ringing cumulative count after mutation.

From Fig. 5, it can be observed that the cumulative count of AE signals experiences a mutation at a certain moment. In this section, the multifractal spectrum before and after the mutation of the cumulative count of AE is calculated, as shown in Fig. 9. It can be seen that the multifractal spectrum before the mutation primarily exhibits a right-hook shape, indicating a similar macroscopic generation mechanism for AE events, where the frequency of small events is higher than that of large events in the AE signal. After the mutation, the multifractal spectrum mainly displays both right-hook and left-hook shapes. A left-hook-shaped multifractal spectrum suggests that the frequency of large events in the AE signal is higher than that of small events. Table 2 provides the multifractal parameters of the cumulative AE signals before and after the mutation for the composite samples. It is evident that the $\Delta \alpha$ values after the mutation are significantly higher than those before the mutation. This indicates that after the mutation, the multifractal characteristics of the signals become more pronounced, the gap between large and small AE signals widens, and the unevenness and fluctuations in the signal distribution become more intense.

**Table 2:** Multifractal parameters of ringing cumulative count before and after mutation.

No	$\Delta \alpha \;({\text{Before}} )$	$\Delta f\left( \alpha \right)\;({\text{Before}} )$	$\Delta \alpha \;({\text{After}} )$	$\Delta f\left( \alpha \right)\;({\text{After}} )$	$\frac{{\Delta \alpha \;({\text{After}} )}}{{\Delta \alpha \;({\text{Before}} )}}$	$\frac{{\Delta f\left( \alpha \right)\;({\text{After}} )}}{{\Delta f\left( \alpha \right)\;({\text{Before}} )}}$
A-1	0.811	0.202	1.186	0.131	1.462	0.649
A-2	0.636	0.226	1.223	0.247	1.922	1.093
A-3	0.292	0.137	1.003	0.076	3.435	0.555
B-1	1.149	0.089	1.406	0.066	1.224	0.742
B-2	0.411	0.262	0.770	0.401	1.873	1.531
B-3	0.331	0.050	0.694	− 0.149	2.097	− 2.980
C-1	0.664	0.213	1.326	0.247	1.997	1.160
C-2	0.447	0.181	1.016	0.343	2.273	1.895
C-3	0.325	0.188	0.922	0.149	2.837	0.793
D-1	0.633	0.249	0.993	0.166	1.569	0.667
D-2	0.409	0.450	0.759	0.526	1.856	1.169
D-3	0.234	0.359	0.697	− 0.220	2.979	− 0.613

**Figure 10:**
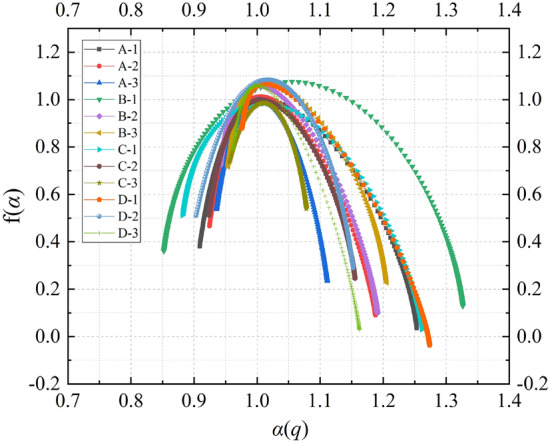
Dynamic multifractal spectrum of low-frequency and high-amplitude signals.

Research has indicated a significant increase in low-frequency, high-amplitude AE signals as a prominent precursor to sample failure^36^. Therefore, this study screened and calculated the multifractal characteristics of the time series of low-frequency, high-amplitude AE signals generated during the loading process of composite samples, as shown in Fig. 10. It is evident from the figure that the multifractal spectra of all composite samples exhibit a left-hook shape, indicating that the frequency of large events in the AE signals is higher than that of small events. This suggests that a large number of microcracks in the composite samples expand and connect to form larger cracks under varying stress conditions. Similarly, a significant increase in low-frequency, high-amplitude signals in the acoustic emission signals indicates the development of microcracks within the specimen evolving into larger fractures, forewarning the imminent failure of the sample. This indirectly verifies the correctness of considering a significant increase in low-frequency, high-amplitude AE signals as a precursor to sample failure. To quantitatively describe the changes in multifractal characteristics of composite samples with different stress loading schemes, Table 3 provides detailed comparative data for the 12 groups of samples. From the table, it can be observed that the values of multifractal parameters are significantly influenced by the mining depths. The overall performance is that the value of the multifractal parameters of the AE time series of the sample loaded by the stress Scheme 3 with the simulated mining depth of 700 m is the smallest, and the value of the multifractal parameters of the AE time series of the sample loaded by the stress Scheme 1 with the simulated mining depth of 500 m is the largest. This shows that the more uniform the AE signal is, the lower the fluctuation of the signal is when the stress scheme with deeper mining depth is used to load the samples.

**Table 3:** Multifractal parameters of low-frequency and high-amplitude signals.

No	$\Delta \alpha$	$\Delta f\left( \alpha \right)$	$\frac{{\Delta \alpha \left( {\text{A - n}} \right)}}{{\Delta \alpha \left( {\text{A - 1}} \right)}}$	$\frac{{\Delta \alpha \left( {\text{B - n}} \right)}}{{\Delta \alpha \left( {\text{B - 1}} \right)}}$	$\frac{{\Delta \alpha \left( {{\text{C}} - {\text{N}} } \right)}}{{\Delta \alpha \left( {\text{C - 1}} \right)}}$	$\frac{{\Delta \alpha \left( {{\text{D}} - {\text{N}} } \right)}}{{\Delta \alpha \left( {\text{D - 1}} \right)}}$
			$\frac{{\Delta f\left( \alpha \right)\left( {\text{A - n}} \right)}}{{\Delta f\left( \alpha \right)\left( {\text{A - 1}} \right)}}$	$\frac{{\Delta f\left( \alpha \right)\left( {\text{B - n}} \right)}}{{\Delta f\left( \alpha \right)\left( {\text{B - 1}} \right)}}$	$\frac{{\Delta f\left( \alpha \right)\left( {\text{B - n}} \right)}}{{\Delta f\left( \alpha \right)\left( {\text{B - 1}} \right)}}$	$\frac{{\Delta f\left( \alpha \right)\left( {\text{B - n}} \right)}}{{\Delta f\left( \alpha \right)\left( {\text{B - 1}} \right)}}$
A-1	0.344	− 0.345	1.000 1.000			
A-2	0.263	− 0.376	0.764 1.090			
A-3	0.176	− 0.303	0.510 0.879			
B-1	0.474	− 0.232		1.000 1.000		
B-2	0.249	− 0.622		0.526 2.687		
B-3	0.252	− 0.489		0.531 2.113		
C-1	0.380	− 0.481			1.000 1.000	
C-2	0.232	− 0.265			0.609 0.551	
C-3	0.122	− 0.196			0.320 0.409	
D-1	0.298	− 0.915				1.000 1.000
D-2	0.249	− 0.220				0.838 0.241
D-3	0.201	− 0.759				0.675 0.829

**Figure 11:**
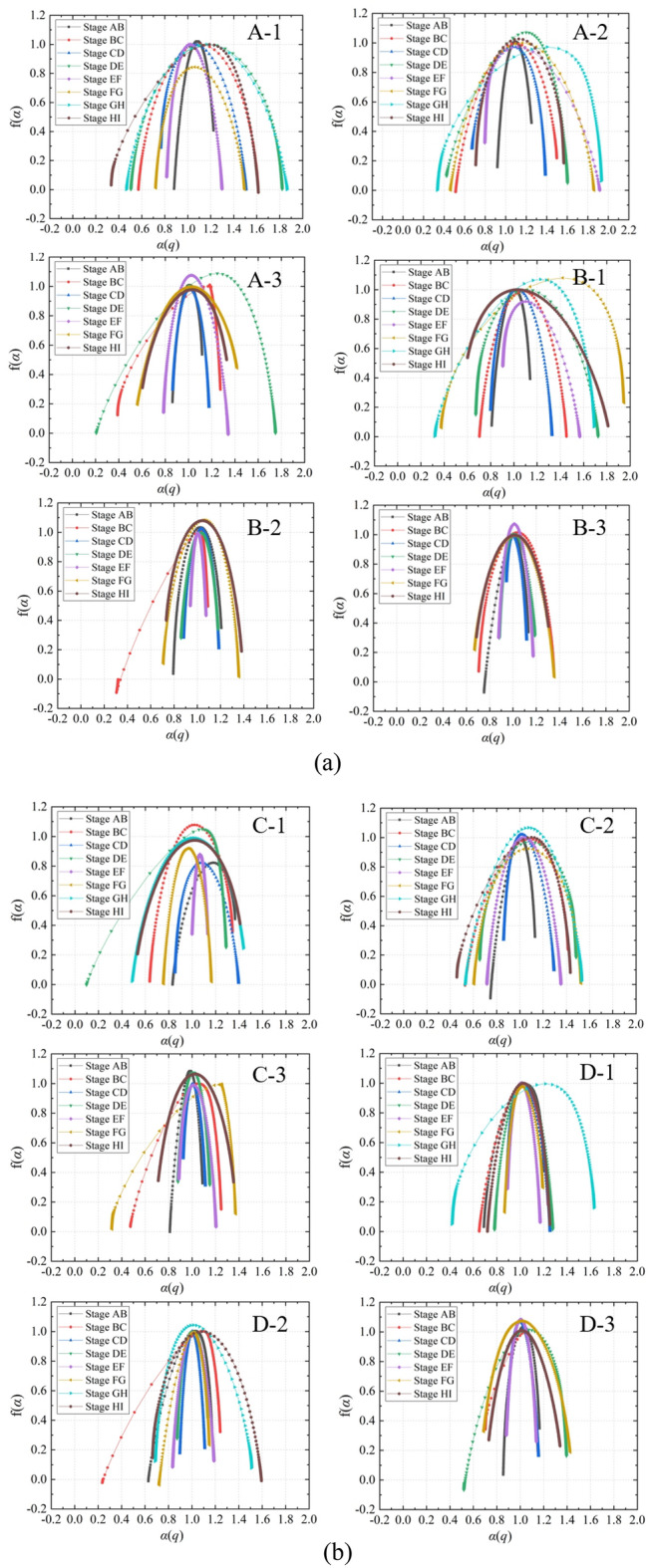
Dynamic multifractal spectrum of different stress stages: (**a**) Three-layer rock structure, (**b**) Four-layer rock structure.

In the A-1 of Fig. 5, the change of stress with time is divided into eight stress stages denoted as A to I. In order to further study the multifractal variation characteristics of each stress loading part, in this part, we calculate the multifractal of AE time series in different stress stages. What needs to be explained here is that in the case of sample A-3, due to its lower inherent strength, the sample has already undergone failure during the decrease in confining pressure, leading to the absence of the GH stage in its stress-time curve. Therefore, the stress-time curve for this sample only includes seven stress stages. Figure 11 presents the multifractal spectra of composite samples during different stress loading and unloading stages. The multifractal spectra in different stages exhibit three main shapes: left-hook, right-hook, and bell-shaped. This indicates that AE events have different macro-generation mechanisms during various stress stages. The left-hook shaped multifractal spectra mainly appear in the CD and EF stages, corresponding to conditions where axial pressure remains constant while confining pressure decreases. Therefore, when the axial pressure is constant and the confining pressure decreases, the frequency of large events in the AE signal is higher than that of small events, that is, due to the pressure relief, a large number of cracks expand and connected.

**Figure 12:**
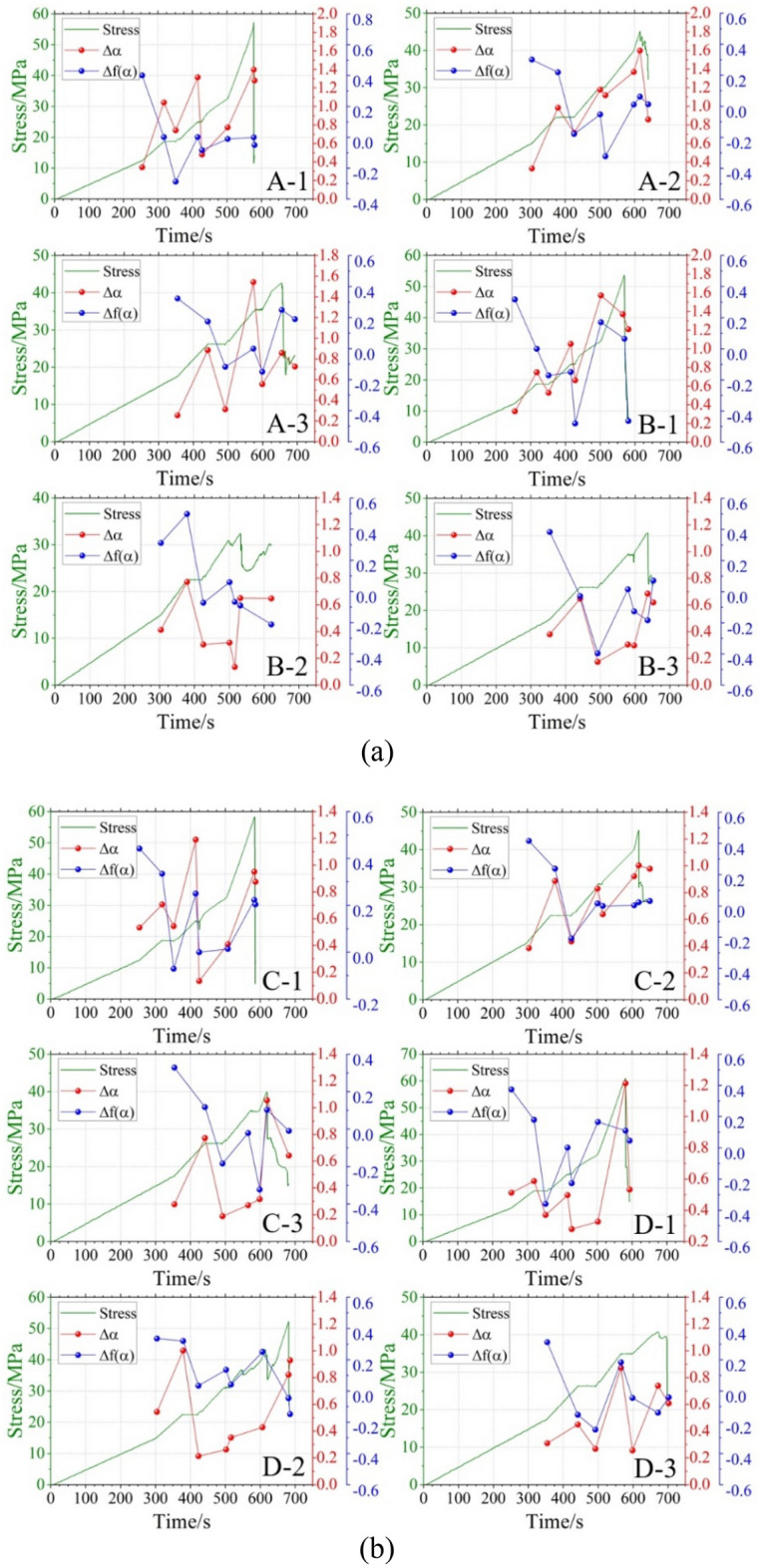
Dynamic multifractal parameters of AE signals in different stages of stress change: (**a**) Three-layer rock structure, (**b**) Four-layer rock structure.

To provide a more intuitive representation of the multifractal characteristics during different stress stages, Fig. 12 combines the multifractal spectrum parameters of the 12 groups of composite samples with the corresponding stress variation curves. Overall, the multifractal spectrum parameter $\Delta \alpha$ shows an increasing trend during the BC, DE, and FG stages. This indicates that when axial pressure increases while confining pressure decreases, the non-uniformity of the AE signal increases, the fluctuation of the signal distribution increases, and some small cracks in the composite sample rapidly expand to form large cracks. In contrast, during the CD and EF stages, $\Delta \alpha$ exhibits a decreasing trend. This suggests that when axial pressure remains constant while confining pressure decreases, the signals become more uniform, and the fluctuation in signal distribution decreases. Combined with the multifractal spectrum parameters $\Delta f\left( \alpha \right)$ of CD and EF segments also showing a decreasing trend, it can be speculated that the reason why the size signal uniformity of CD and EF stages increases is that the cracks develop rapidly at the stages, and the AE signals are mainly large signals. Furthermore, during the HI stage (residual stress stage), most composite samples show a decreasing trend in both $\Delta \alpha$ and $\Delta f\left( \alpha \right)$, which also shows that the AE signals are mainly large signal, and the samples are instability and failure.

The $\Delta \alpha$ generally shows an increasing trend from the AB stage (hydrostatic pressure stage) to the HI stage (residual stress stage), while $\Delta f\left( \alpha \right)$ exhibits a decreasing trend. This indicates that, compared to the stress initial stage, the unevenness of the AE signals increases during the residual stress stage, with a higher proportion of large signals. The microcrack process in the composite samples becomes more complex during the residual stress stage. To quantitatively describe the changes in multifractal characteristics between the hydrostatic pressure stage and the residual stress stage, Table 4 provides detailed comparative data. In the table, $\Delta \alpha \left( {{\text{HI}} - {\text{AB}}} \right)$ represents the difference between the $\Delta \alpha$ value in the HI stage and the $\Delta \alpha$ value in the AB stage, and $\Delta f\left( \alpha \right)\left( {{\text{HI}} - {\text{AB}}} \right)$ represents the difference between the $\Delta f\left( \alpha \right)$ value in the HI stage and the $\Delta f\left( \alpha \right)$ value in the AB stage. These values indicate the changes in AE signals before and after loading and unloading of the composite samples. Overall, the change in the three-layer rock sample (Groups A and B) is higher than that in the four-layer rock sample (Groups C and D), indicating a more intense destruction in the three-layer sample group during the loading and unloading process. In the AB stage, the $\Delta \alpha$ value of the three-layer rock composite samples varies between 0.255 and 0.413, with a range of 0.158, and the $\Delta f\left( \alpha \right)$ value varies between 0.301 and 0.401, with a range of 0.100. In the AB stage, the $\Delta \alpha$ value of the four-layer rock composite samples varies between 0.278 and 0.544, with a range of 0.266, and the $\Delta f\left( \alpha \right)$ value varies between 0.312 and 0.442, with a range of 0.130. The range of variation in the two parameters for the four-layer rock composite samples is larger than that for the three-layer rock composite samples, indicating that during the hydrostatic pressure stage, the three-layer rock composite samples have relatively uniform AE signals, stable signal distribution, and a small difference in the proportion of large and small signals. The increased complexity in the microcrack distribution of the four-layer rock samples may be due to the additional bonding steps in their preparation, leading to increased signal unevenness in the samples. Therefore, by calculating the multifractal parameters of AE during the hydrostatic pressure stag in composite samples, samples with relatively discrete $\Delta \alpha$ and $\Delta f\left( \alpha \right)$ values can be identified. This can effectively eliminate poorly prepared samples, reducing experimental errors.

**Table 4:** Multifractal dynamic change parameter of different composite samples.

No	$\Delta \alpha ({\text{AB}} )$	$\Delta \alpha ({\text{HI}} )$	$\Delta f\left( \alpha \right)({\text{AB}} )$	$\Delta f\left( \alpha \right)({\text{HI}} )$	$\Delta \alpha \left( {{\text{HI}} - {\text{AB}}} \right)$	$\Delta f\left( \alpha \right)\left( {{\text{HI}} - {\text{AB}}} \right)$
A-1	0.343	1.280	0.401	− 0.050	0.937	− 0.451
A-2	0.333	0.858	0.301	0.013	0.525	− 0.288
A-3	0.255	0.726	0.324	0.191	0.471	− 0.133
B-1	0.331	1.209	0.317	− 0.464	0.878	− 0.781
B-2	0.413	0.648	0.312	− 0.211	0.235	− 0.523
B-3	0.381	0.620	0.384	0.071	0.239	− 0.313
C-1	0.534	0.876	0.442	0.204	0.342	− 0.238
C-2	0.384	0.977	0.417	0.031	0.593	− 0.386
C-3	0.278	0.643	0.328	− 0.010	0.365	− 0.338
D-1	0.513	0.533	0.374	0.046	0.020	− 0.328
D-2	0.544	0.930	0.336	− 0.148	0.386	− 0.484
D-3	0.310	0.608	0.312	− 0.041	0.298	− 0.353

## Conclusions

In this paper, three sets of triaxial loading and unloading experimental schemes for simulating the actual stress distribution in coal mining sites are designed. The deformation and failure of composite samples with different stress loading and unloading schemes and the dynamic multifractal characteristics of AE time series are discussed. The main conclusions are as follows:

- (1) The composite samples loaded by simulating different mining depth stress schemes show a decreasing trend with the increase of mining depth. And the strength of the composite sample of the four-layer rock layer loaded by the large mining depth stress scheme is lower than that of the composite sample of the three-layer rock layer structure, while the strength of the four-layer rock layer loaded by the low mining depth stress scheme is higher than that of the three-layer rock layer structure. This shows that with the increase of coal seam mining depth, the bearing capacity of different rock strata structures will change.
- (2) The multifractal spectrum parameter $\Delta \alpha$ of the AE time series when the composite sample is loaded by simulating the stress scheme of different mining depths shows a trend of smaller $\Delta \alpha$ value with the increase of mining depth, while the change rule of $\Delta f\left( \alpha \right)$ shows a trend of larger $\Delta f\left( \alpha \right)$ value with the increase of mining depth. The change degree of multifractal parameters of composite samples with four-layer rock structure is lower than that of composite samples with three-layer rock structure with different loading schemes. The micro-fracture process of three-layer composite samples is more complicated and the degree of damage is higher. Researchers can extend the study to rocks of different lithologies to assess variations in their performance within composite structures. This can provide research outcomes with greater applicability to a broader range of geological conditions.
- (3) The value of the multifractal spectrum parameter $\Delta \alpha$ of the time series after the mutation of the cumulative count of the AE signal is much larger than the $\Delta \alpha$ value before the mutation, which indicates that the multifractal characteristics of the signal after the mutation are more obvious. The difference between the AE signals increases, the non-uniformity among the signals intensifies, and the fluctuation of the signal distribution becomes more pronounced.
- (4) The dynamic changes in the multifractal parameters $\Delta \alpha$ and $\Delta f\left( \alpha \right)$ at different stress loading and unloading stages reflect the influence of axial pressure or confining pressure variations on the expansion of cracks in composite samples. Overall, $\Delta \alpha$ shows an increasing trend from the hydrostatic pressure stage to the residual stress stage, while $\Delta f\left( \alpha \right)$ exhibits a decreasing trend. This indicates that, compared to the initial stress stage, the non-uniformity of AE signals increases in the residual stress stage, and the proportion of large signals becomes more prominent, signifying a complex micro-fracture process in the composite samples.

## Data Availability

The datasets used and/or analyzed during the current study available from the corresponding author on reasonable request.
